# Error-prone initiation factor 2 mutations reduce the fitness cost of antibiotic resistance

**DOI:** 10.1111/j.1365-2958.2010.07057.x

**Published:** 2010-02-03

**Authors:** Anna Zorzet, Michael Y Pavlov, Annika I Nilsson, Måns Ehrenberg, Dan I Andersson

**Affiliations:** 1 Department of Medical Biochemistry and Microbiology, Uppsala UniversityBox 582, SE-751 23 Uppsala, Sweden; 2 Department of Cell and Molecular Biology, Uppsala UniversityBox 596, SE-751 24 Uppsala, Sweden

## Abstract

Mutations in the *fmt* gene (encoding formyl methionine transferase) that eliminate formylation of initiator tRNA (Met-tRNA_i_) confer resistance to the novel antibiotic class of peptide deformylase inhibitors (PDFIs) while concomitantly reducing bacterial fitness. Here we show in *Salmonella typhimurium* that novel mutations in initiation factor 2 (IF2) located outside the initiator tRNA binding domain can partly restore fitness of *fmt* mutants without loss of antibiotic resistance. Analysis of initiation of protein synthesis *in vitro* showed that with non-formylated Met-tRNA_i_ IF2 mutants initiated much faster than wild-type IF2, whereas with formylated fMet-tRNA_i_ the initiation rates were similar. Moreover, the increase in initiation rates with Met-tRNA_i_ conferred by IF2 mutations *in vitro* correlated well with the increase in growth rate conferred by the same mutations *in vivo*, suggesting that the mutations in IF2 compensate formylation deficiency by increasing the rate of *in vivo* initiation with Met-tRNA_i_. IF2 mutants had also a high propensity for erroneous initiation with elongator tRNAs *in vitro*, which could account for their reduced fitness *in vivo* in a formylation-proficient strain. More generally, our results suggest that bacterial protein synthesis is mRNA-limited and that compensatory mutations in IF2 could increase the persistence of PDFI-resistant bacteria in clinical settings.

## Introduction

Initiation of protein synthesis takes place after splitting the post-termination 70S ribosome into its small (30S) and large (50S) subunits. The ribosome splitting, which is catalysed by ribosome recycling factor (RRF) and elongation factor G (EF-G), is followed by the binding of initiation factor 3 (IF3) to the 30S subunit (Karimi *et al.*, 1999; Peske *et al.*, 2005; Pavlov *et al.*, 2008). Subsequent binding of initiation factors 1 (IF1) and 2 (IF2), messenger RNA (mRNA) and initiator tRNA (formyl(f)-Met-tRNA_i_) to the 30S:IF3 complex results in the formation of the 30S pre-initiation complex (30S PIC) (Gualerzi *et al.*, 2001; Antoun *et al.*, 2006a).

Correct positioning of the start codon of mRNAs in the P site of the 30S subunit requires the presence of fMet-tRNA_i_ (rather than an elongator tRNA) in the 30S PIC (Hartz *et al.*, 1989). The accuracy of initiator tRNA selection into the 30S PIC is greatly enhanced by IF1, IF2 and IF3 (Wintermeyer and Gualerzi, 1983; Pon and Gualerzi, 1984; Canonaco *et al.*, 1986; Antoun *et al.*, 2006a,b;). IF3 blocks premature docking of the 50S subunit to an initiator tRNA-less 30S PIC and increases the rate constants for tRNA association to, and dissociation from, the 30S subunit (Subramanian and Davis, 1970; Gualerzi *et al.*, 2001; Lancaster and Noller, 2005; Antoun *et al.*, 2006a). IF2 plays a pivotal role in the fast binding of fMet-tRNA_i_ into the 30S PIC (Benne *et al.*, 1973; Fakunding and Hershey, 1973; Wintermeyer and Gualerzi, 1983; Gualerzi *et al.*, 2001; Antoun *et al.*, 2006a) and the subsequent rapid docking of the 50S subunit to the 30S PIC containing fMet-tRNA_i_ (Antoun *et al.*, 2003; 2006a; Grigoriadou *et al.*, 2007), thereby ensuring high accuracy of initiator tRNA selection into the 70S initiation complex (Antoun *et al.*, 2006b). IF1 together with IF2 and IF3 enhances the accuracy of initiator tRNA selection by selectively increasing the rate of fMet-tRNA_i_ binding to the 30S subunit (Pon and Gualerzi, 1984; Antoun *et al.*, 2006b).

The formyl group of fMet-tRNA_i_ greatly increases the ability of IF2 to distinguish between initiator tRNA and aminoacylated elongator tRNAs (Sundari *et al.*, 1976; Gualerzi *et al.*, 2001; Antoun *et al.*, 2006b). Formylation of Met-tRNA_i_ is catalysed by the formyl-methionine-transferase (FMT), which recognizes a C1·A72 mismatch present in tRNA_i_ but absent in most bacterial elongator tRNAs (RajBhandary, 1994). Formylation of the methionine of initiator tRNA and subsequent removal of the formyl group from finished proteins by peptide deformylase (PDF) occur in most eubacteria as well as in mitochondria and chloroplasts of eukaryotes (Solbiati *et al.*, 1999; Vaughan *et al.*, 2002). Deformylation of formylated proteins by PDF is often required for their activity (Solbiati *et al.*, 1999; Bingel-erlenmeyer *et al.*, 2008), making this enzyme an attractive target for novel antimicrobial drugs (Vaughan *et al.*, 2002). One example is PDF inhibitors such as actinonin that cause accumulation of non-functional formylated proteins in the cell, ultimately leading to arrested cell growth (Chen *et al.*, 2000).

Mutations abolishing formylation of Met-tRNA_i_ confer resistance to actinonin since bypass of the formylation step makes the deformylation step and hence the PDF activity redundant (Apfel *et al.*, 2001). Formylation deficiency in *Enterobacteriaceae* leads to very slow growth, showing that formylation is required for fast growth but not for cell viability (Guillon *et al.*, 1992; 1996; Steiner-Mosonyi *et al.*, 2004). However, resistant bacteria can reduce the fitness cost associated with formylation deficiency by acquiring compensatory mutations at maintained resistance (Andersson and Levin, 1999; Andersson, 2006). Second-site mutations that increase the growth rate of formylation-deficient bacteria have previously been found (Margolis *et al.*, 2000; Nilsson *et al.*, 2006). For example, in *S. typhimurium* lack of a functional FMT enzyme can be efficiently compensated by increasing the Met-tRNA_i_ concentration via high-level copy-number amplification of the tRNA_i_ genes *metZ* and *metW* (Nilsson *et al.*, 2006).

Here we identified novel mutations in IF2 that partially compensated for the formylation deficiency in actinonin resistant strains with a normal concentration of initiator tRNA. IF2 consists of four structural domains (Roll-Mecak *et al.*, 2000), with the initiator tRNA-binding domain IV connected to domain III by a long helical linker (Roll-Mecak *et al.*, 2000; Allen *et al.*, 2005; Simonetti *et al.*, 2008). Previously, it was demonstrated that the activity of IF2 in initiation with non-formylated Met-tRNA_i_ can be increased by specifically engineered mutations in domain IV that increase the affinity of IF2 for Met-tRNA_i_ (Steiner-Mosonyi *et al.*, 2004). Unexpectedly, none of the IF2 compensatory mutations identified here were located in domain IV, indicating that they do not compensate the formylation deficiency by increasing the IF2 affinity for Met-tRNA_i_. Instead, this new class of IF2 mutants appear to increase the rate of initiation with Met-tRNA_i_ by increasing the propensity of IF2 to adopt the 50S docking conformation on the 30S ribosomal subunit not only in the presence of fMet-tRNA_i_, but also with Met-tRNA_i_, deacylated tRNA_i_ and elongator tRNAs.

We propose that the IF2 mutants with the strongest compensatory effect have reduced growth rates in a formylation-proficient background due to a highly increased frequency of aberrant initiation events. Importantly, from the observed linear correlation between our biochemical data on the initiation time and the measured bacterial generation time, we suggest that the rate of bulk protein synthesis in the cell is mRNA limited, leading to hypersensitive variation in growth rate in response to variation in initiation rate.

## Results

### *In vivo* analysis

**Fig. 1:**
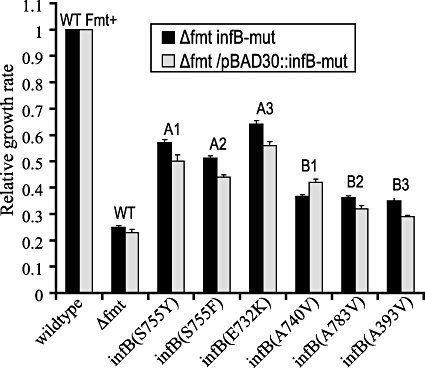
Comparison of growth rates for the formylation-deficient strains with compensatory IF2 mutations, original Fmt^-^ strain and formylation-proficient Fmt^+^ wild-type strain. Black bars represent strains harbouring the IF2 mutation on the chromosome, light grey bars represent the IF2-mutants on plasmid pBAD30. Mutants are grouped as class A (strongly compensating) and class B (weakly compensating).

We previously subjected five different formylation-deficient and slow-growing actinonin-resistant *fmt* mutants to compensatory evolution to select for mutants with increased growth rate (Nilsson *et al.*, 2006). After 50–150 generations of growth, fast-growing mutants were recovered and their compensatory mutations were identified as either point mutations in *fmt*, amplification of the tRNA_i_ genes *metZ* and *metW* or as an unknown class of mutations (Nilsson *et al.*, 2006). In the present study we have identified the compensatory mutations in the latter class as point mutations in the *infB* gene, coding for initiation factor 2 (IF2). Transfer of the five unique point mutations into a formylation-deficient (*fmt* mutant) strain confirmed their growth compensatory nature (Table 1; Fig. 1).

**Table 1:** IF2 mutants isolated after compensatory evolution or localized hydroxylamine mutagenesis (see *Experimental procedures*).

Strain number	Mutation	Location in IF2 structure	IF2 mutant
Compensatory evolution			
**DA10610**	**A740V, gcg→gtg**	**Domain III, H10**	**B1**
DA8781	S741F, tcc→ttc	Domain III, H10	
DA8836	R751L, cgt→ctt	Domain III	
**DA10710**	**A783V, gcg→gtg**	**Linker, H12**	**B2**
**DA10609**	**S755Y, tct→tat**	**Domain III, H11**	**A1**
Hydroxylamine mutagenesis			
DA13317	A740V, gcg→gtg	Domain III, H10	(B1)^a^
DA13318	A783T, gcg→acg	Linker, H12	
DA13363	A182T, gct→act	N-Domain	
**DA13368**	**A393V, gcc→gtc**	**G-domain**	**B3**
DA13369	E763K, gaa→aaa	Domain III	
**DA14394**	**E732K, gaa→aaa**	**Domain III, H10**	**A3**
DA14395	A752T, gcc→acc	Domain III	
**DA14396**	**S755F, tct→ttt**	**Domain III, H11**	**A2**
DA14397	A484V, gct→gtt	G-domain, H4	

a This mutation was found twice.

Both the nucleotide change and the resulting amino acid change are shown. Mutant strains further tested *in vitro* are marked in bold. Location of the mutations in IF2 structure follows the nomenclature used in Roll-Mecak *et al.* (2000).

**Fig. 2:**
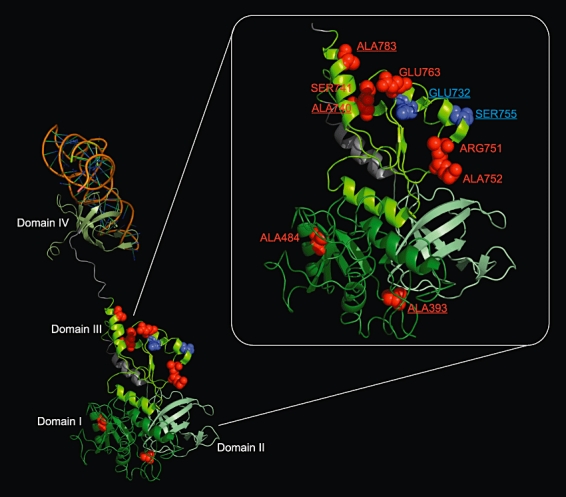
The different point mutations isolated in this study highlighted in the structure of IF2. Class A mutations are marked in blue, other mutations are marked in red and *in vitro* tested IF2 mutations are underlined. The A182T mutation is not shown beacouse the structure does not contain the N-terminal of the protein. The domain nomenclature is the same as in (Roll-Mecak *et al.*, 2000). The structure is based on PDB file 1ZO1 and rendered with the program PyMOL (DeLano, 2002; http://www.pymol.org)

We then extended the search for growth compensating IF2 mutants by performing localized hydroxylamine mutagenesis of *infB*. Phage P22 grown on a strain with a transposon inserted near *infB* was isolated, mutagenized with hydroxylamine and used to transduce a slow-growing *fmt* mutant strain. By screening for fast growers among the tetracycline resistant transductants, nine individual compensated mutants were isolated and their *infB* genes were sequenced. Eight novel *infB* mutations were found (Table 1) and as eight out of the nine mutations were recovered only once, this indicates that the mutational target is not saturated with compensatory mutations. In total, 13 different IF2 mutations, all located well outside tRNA binding domain IV of IF2, have been identified (Table 1; Fig. 2). Cryo-EM studies show also that none of the IF2 mutations could have any direct contact with fMet-tRNA_i_ in the 30S pre-initiation and the 70S initiation complexes (Allen *et al.*, 2005; Myasnikov *et al.*, 2005; Simonetti *et al.*, 2008). It is therefore highly unlikely that any of the mutations in IF2 isolated here conferred growth compensation simply by increasing the affinity of IF2 to Met-tRNA_i_.

### Growth rates of IF2 mutants in a formylation-deficient background

We used linkage to a nearby transposon to transfer six different IF2 mutations into the same *fmt* mutant background to compare the growth rate in rich medium for each one of the different *infB* mutations with the wild-type in an isogenic strain background. The tested mutants could be roughly divided into two classes, one with high (A1–A3) and one with low (B1–B3) compensatory effect. Figure 2 and Table 1 show that the class A compensatory mutations are located in neighbouring helices H10 (A3) and H11 (A1 and A2) of the domain III, while the class B mutations are located in domain III (B1), in the helical linker between domains III and IV (B2) and in the G-domain (B3) of IF2. The bacteria in both classes grew faster than the original *fmt* mutant with wild-type IF2 (Fig. 1).

To further verify that the *infB* mutations and resulting amino acid substitutions in IF2 were solely responsible for the observed fitness-increasing phenotype, a complementation test was performed. The *infB* mutant genes were cloned into the vector pBAD30, transformed into the *fmt* mutant strain and growth rates were measured. As expected, growth rates were improved but were slightly lower than the previously measured mutant growth rates (Fig. 1). This small reduction in the compensatory effect of the mutations could be due to the presence of wild-type IF2 in these complemented strains.

### Growth rates of IF2 mutants in a formylation-proficient background

**Fig. 3:**
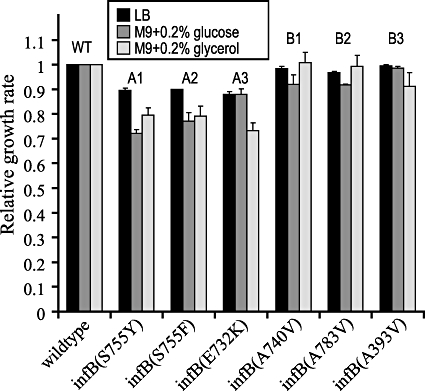
Fitness costs of the IF2 mutations in an otherwise wild-type formylation-proficient background (Fmt^+^). Growth rates were measured in LB media (black bars) and M9 minimal media with either 0.2% glucose (grey bars) or glycerol (light grey bars) as the carbon source.

We compared the growth rate of a strain with wild-type IF2 to the growth rates of the IF2 mutants in an otherwise wild-type background in rich media and in minimal media supplemented with either glucose or glycerol. The class A IF2 mutants grew 10–25% slower than the wild-type strain, whereas the class B mutants showed a smaller (< 5%) growth rate reduction (Fig. 3). For both mutant classes, the fitness reduction was higher in poor than in rich media (Fig. 3). To confirm that the fitness effects seen *in vivo* resulted from altered activity of the mutant IF2 rather than from alterations in the intracellular level of mutant protein, we measured IF2 levels during exponential growth using Western blotting. No difference in the steady-state level of IF2 could be seen between the wild-type, the parental *fmt* mutant and the various IF2 mutants (data not shown).

### *In vitro* analysis

Two steps in initiation of translation depend crucially on IF2: (i) binding of fMet-tRNA_i_ to the mRNA-containing 30S subunit and (ii) subsequent docking of the 50S subunit to the complete 30S PIC (Hartz *et al.*, 1991; Gualerzi *et al.*, 2001; Antoun *et al.*, 2006a,b;). These two steps of initiation were mimicked in our biochemical experiments by rapidly mixing 50S subunits and initiator tRNAs with tRNA-lacking 30S PICs in a stopped flow instrument and monitoring the formation of 70S initiation complexes by Rayleigh light scattering (Antoun *et al.*, 2003). The experiments were performed with wild-type IF2 and different IF2 mutants in combination with formylated, non-formylated or deacylated initiator tRNA as well as an aminoacylated elongator tRNA.

### Initiation with IF2 mutants and formylated initiator tRNA

**Fig. 4:**
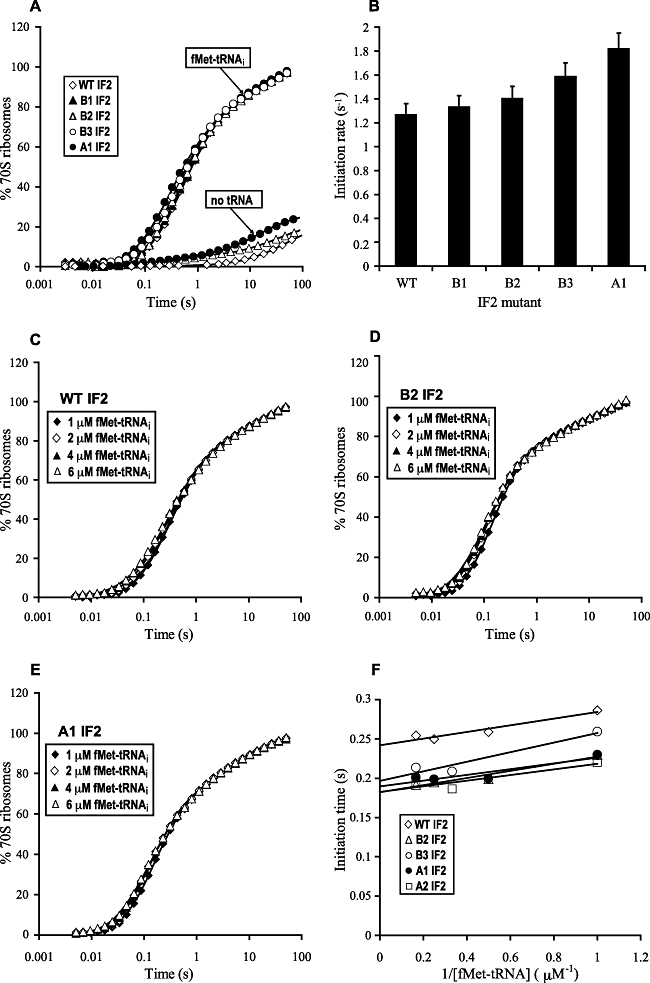
Kinetics of 70S initiation complex formation after rapid mixing of tRNA-free 30S PICs containing different IF2s with 50S subunits and fMet-tRNA_i_ and their dependence on fMet-tRNA_i_ concentration. A. 30S PICs assembled with 1 µM IF3 were mixed with 50S subunits and 0.5 µM fMet-tRNA_i_. B. Rates (*k*_I_) of 70S initiation complex formation in the experiments shown in (A). C. 30S PICs assembled with 0.5 µM IF3 were mixed with 50S subunits and fMet-tRNA_i_ in 1, 2, 4 or 6 µM concentration. D. The same as (C) but 30S complexes contained B2 IF2 mutant. E. The same as (C) but 30S complexes contained A1 IF2 mutant. F. L-B plot of the dependence of initiation time *t*_0.5_ (= 1/*k*_I_) on the inverse of fMet-tRNA_i_ concentration for different IF2 variants. All concentrations in the figures are given as final concentrations after the mixing.

Formation of 70S initiation complex after rapid mixing of 50S subunits and formylated initiator Met-tRNA_i_ (fMet-tRNA_i_) with 30S PICs lacking tRNA proceeded with similar rates for wild-type and IF2 mutants (Fig. 4A). In the absence of initiator tRNA the rate and extent of 70S complex formation were very small, although significantly larger for class B mutants than for wild-type and significantly larger for class A than for class B mutants of IF2 (Fig. 4A). For simple quantification of the initiation rate (*k*_I_), which involves initiator tRNA binding to active 30S PICs, subsequent docking of 50S subunits to tRNA-containing active 30S PICs and a slow conversion of a small fraction of the 30S PICs inactive in 50S docking into active complexes (Milon *et al.*, 2008), we defined *k*_I_ as the inverse of the time, t_0.5_, at which 50% of the 70S initiation complexes have been formed after mixing (see *Experimental procedures*). The initiation rate, *k*_I_, was lowest for wild-type IF2 (1.3 s^−1^) and highest for the A1 mutant (1.8 s^−1^) (Fig. 4B). It varied but little with increasing initiator tRNA concentration above 1 µM (Fig. 4C–E, Fig. S1). Lineweaver–Burk (L-B) plots of the dependence of the initiation time, 1/*k*_I_, on the inverse of fMet-tRNA_i_ concentration (Fig. 4F) show that the minimal initiation time obtained by extrapolation to saturating fMet-tRNA_i_ concentration varied from 0.18 s for A1 IF2 mutant up to 0.24 s for wild-type IF2. These minimal times correspond to maximal initiation rates of 5.5 and 4.1 s^−1^ for the A1 mutant and wild-type IF2 respectively. Note that higher IF3 concentration in experiments in Fig. 4A (1 µM IF3) than in Fig. 4C–E (0.5 µM IF3) accounts for a slower initiation in the former case (Antoun *et al.*, 2006a).

### Initiation with IF2 mutants and un-formylated Met-tRNA_i_

**Fig. 5:**
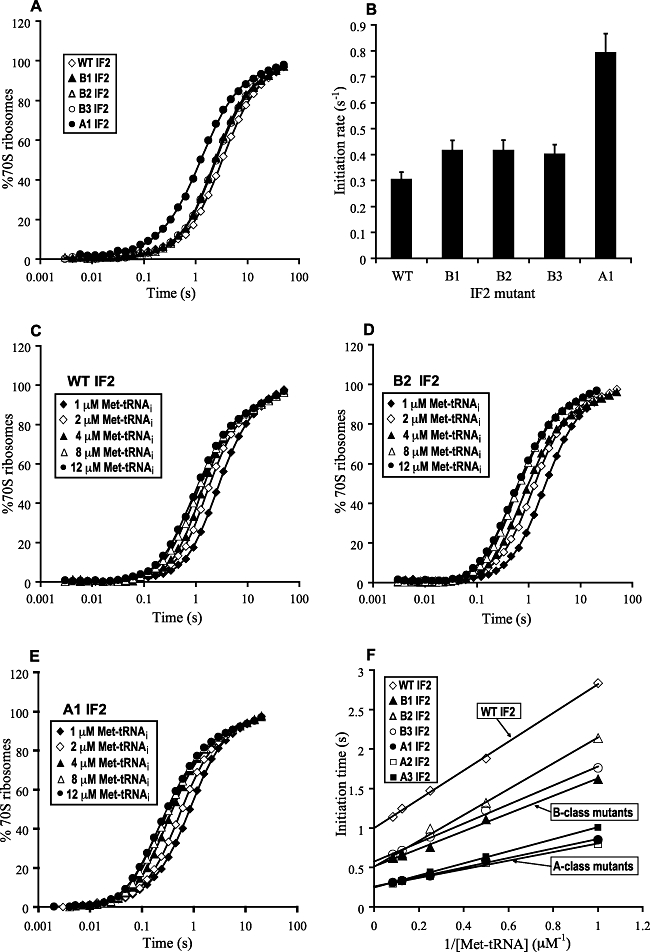
Kinetics of 70S initiation complex formation after rapid mixing of tRNA-free 30S PICs containing different IF2s with 50S subunits and non-formylated Met-tRNA_i_ and their dependence on Met-tRNA_i_ concentration. A. 30S PICs were mixed with 50S subunits and 0.8 µM Met-tRNA_i_. B. Rates (*k*_I_) of 70S initiation complex formation for the experiments in (A). C. The same as in (A) but Met-tRNA_i_ was added in 1, 2, 4, 8 or 12 µM concentration with 50S subunits and 30S PICs contained WT IF2. D. The same as (C) but 30S PICs contained B2 IF2 mutant. E. The same as (C) but 30S PICs contained A1 IF2 mutant. F. L-B plot of the dependence of initiation time t_0.5_ (= 1/*k*_I_) on the inverse of Met-tRNA_i_ concentration for all IF2 variants.

The rates of initiation in experiments where 50S subunits and un-formylated Met-tRNA_i_ were mixed with tRNA-lacking 30S PICs (Fig. 5A) were significantly lower than the initiation rates with formylated fMet-tRNA_i_ (compare Fig. 4B with Fig. 5B). In addition, the rates obtained with Met-tRNA_i_ displayed much larger relative differences between the different IF2 variants, with an almost threefold larger initiation rate for the A1 IF2 mutant than for wild-type IF2 (Fig. 5B).

Initiation with Met-tRNA_i_ also depended strongly on the initiator tRNA concentration for all IF2 variants (Fig. 5C–E, Fig. S2), in contrast to initiation with fMet-tRNA_i_ (compare Fig. 4F with Fig. 5F). The slow Met-tRNA_i_ binding to the 30S PIC at low Met-tRNA_i_ concentration is seen in Fig. 5 as a pronounced time-delay in the formation of 70S initiation complexes after the start of the initiation reaction.

The L-B plots (Fig. 5F) of the dependence of the initiation time, *1*/*k*_I_, on the inverse of the Met-tRNA_i_ concentration determine the sensitivity (*k*_max_/*K*_M_) of the initiation rate, *k*_I_, to the Met-tRNA_i_ concentration and the maximal initiation rate in the limit of saturating Met-tRNA_i_ concentration (*k*_max_). In the presence of either one of the A-IF2 or B-IF2 mutants the *k*_max_-values were around 4 or 2 s^−1^, respectively, whereas with wild-type IF2, *k*_max_ was about 1 s^−1^ (Table 2). Although both *k*_max_/*K*_M_ and *k*_max_ were increased by the IF2 mutations, the increase was larger in *k*_max_ than in *k*_max_/*K*_M_ (Table 2), showing that the increased rate of 50S docking to the 30S subunit was the predominant effect of these mutations (see also Supporting information).

**Table 2:** Kinetic parameters for 70S initiation complex formation upon addition of 50S subunits and Met-tRNA_i_ to tRNA-lacking 30S PICs.

IF2 mutant	*k*_max_	*k*_max_/*K*_M_	*K*_M_
WT	1.01 ± 0.03	0.55 ± 0.02	1.82 ± 0.06
B1	1.95 ± 0.12	0.90 ± 0.05	2.17 ± 0.18
B2	1.96 ± 0.18	0.61 ± 0.03	3.21 ± 0.36
B3	1.74 ± 0.09	0.83 ± 0.05	2.09 ± 0.15
A1	3.89 ± 0.31	1.59 ± 0.11	2.44 ± 0.22
A2	3.87 ± 0.21	1.83 ± 0.09	2.12 ± 0.16
A3	4.06 ± 0.12	1.31 ± 0.03	3.11 ± 0.09

**Fig. 6:**
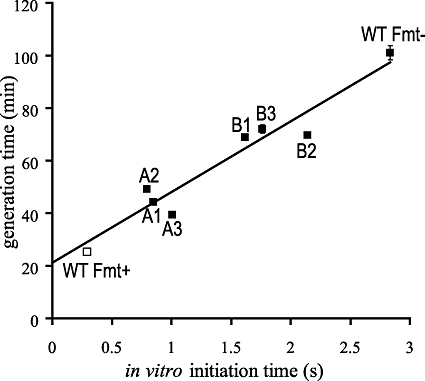
Correlation between generation times of *fmt* mutant strains harbouring different IF2s and wild-type and the *in vitro* initiation times measured with 1 µM Met-tRNA_i_ added together with 50S subunits to tRNA-free 30S PIC containing corresponding IF2s.

The L-B plots also show how the initiation time, *1*/*k*_I_, varied for different IF2s at a fixed concentration of Met-tRNA_i_ (Fig. 5F). Remarkably, when the initiation time was plotted against the generation time of *fmt*-deficient strains harbouring these IF2 mutations, a strong linear correlation was observed (Fig. 6). The correlation was robust to changes in Met-tRNA_i_ concentration in the 1–2 µM range (Fig. S3), corresponding to the estimated *in vivo* range for the free concentration of initiator tRNA (Gualerzi and Pon, 1990). Notably, the time of about 0.25 s for *in vitro* initiation with fMet-tRNA_i_ (Fig. 4F) and the generation time for the wild-type strain (25.2 min in Fig. 6) were on the same straight line as the mutant points (see *Discussion*).

### IF2 mutants cause aberrant 70S initiation complex formation

*In vivo*, a formylation-proficient strain harbouring any one of the class A IF2 mutants grew slower than wild-type under all tested growth conditions (Fig. 3). In contrast, *in vitro* initiation with authentic formylated initiator tRNA proceeded faster with class A IF2 mutants than with wild-type IF2 (Fig. 4B and 4F), which suggested that the growth rate reduction with class A IF2 mutants *in vivo* had other reasons than impaired mainstream initiation.

**Fig. 7:**
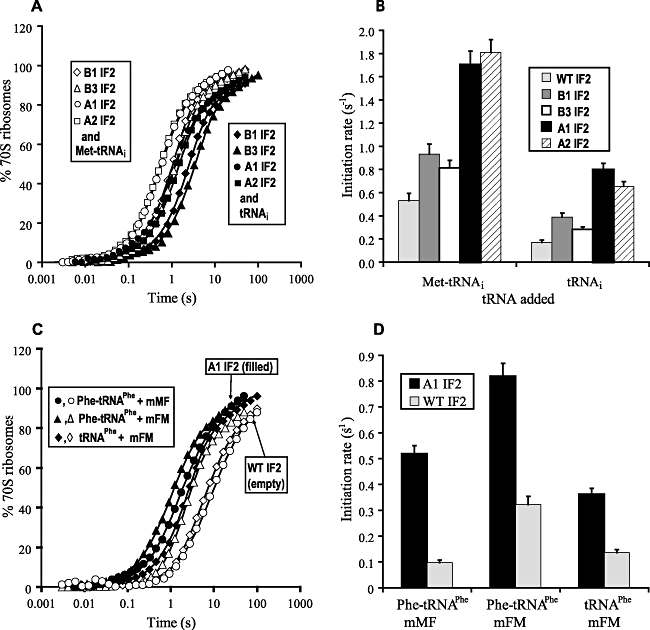
Kinetics of the formation of initiation 70S complexes with Met-tRNA_i_, abortive 70S complexes with deacylated tRNA_i_ or tRNA^Phe^ and aberrant 70S complexes with Phe-tRNA^Phe^. A. 30S PICs were mixed with 50S subunits and 2 µM deacylated tRNA_i_ or methionylated Met-tRNA_i_. B. Rates (*k*_I_) of 70S formation for the experiments in (A). C. Deacylated tRNA^Phe^ or Phe-tRNA^Phe^ in 2 µM concentration were added together with 50S subunits to 30S PICs assembled with 0.8 µM of mMFTI (mMF) or mFMTI (mFM) mRNA. D. Rates (*k*_I_) of 70S formation for the experiments in (C).

To identify these reasons, we first studied the formation of an ‘abortive’ 70S initiation complex (i.e. a complex unable to provide the donor in peptidyl-transfer) after rapid mixing of 50S subunits plus deacylated tRNA_i_ with tRNA-lacking 30S PICs (Fig. 7A). At a final tRNA_i_ concentration of 2 µM, the rate of abortive 70S initiation complex formation was 0.15 s^−1^ for wild-type, approximately 0.35 s^−1^ for B-and 0.75 s^−1^ for the class A IF2 mutants (Fig. 7B).

Next, we studied the rate of formation of aberrant 70S initiation complexes, containing the aminoacylated form of the elongator tRNA^Phe^ in the presence of wild-type IF2 or the A1 mutant. With mRNA where the initiation codon was optimally positioned in relation to the Shine–Dalgarno sequence, the rate of aberrant 70S complex formation with Phe-tRNA^Phe^ was fivefold higher in the presence of the A1 mutant than the wild-type IF2 (Fig. 7C and D). Taking into account that the formation of 70S initiation complex with fMet-tRNA_i_ proceeded similarly for all IF2s (Fig. 4F), the experiments in Fig. 7 suggest that, at a given free concentration of deacylated tRNA_i_ or acylated elongator tRNA in the cell, the class A mutants of IF2 would cause a fivefold higher frequency of formation of abortive or aberrant 70S initiation complex.

Figure 7C and D also shows that swapping of the Phe and initiation codons in mRNA, placing the former in the optimal position in relation to the Shine–Dalgarno sequence, increased the rate of aberrant 70S complex formation about 50% with the A1 mutant IF2 and about threefold with the wild-type IF2, reducing the effect of the A1 mutation in IF2 on initiation with Phe-tRNA^Phe^ (or deacylated tRNA^Phe^) to about threefold (Fig. 7C and D).

### The A1 IF2 mutation decreases the effect of tRNA_i_ acylation and formylation on the rate of 50S docking to the 30S pre-initiation complex

**Fig. 8:**
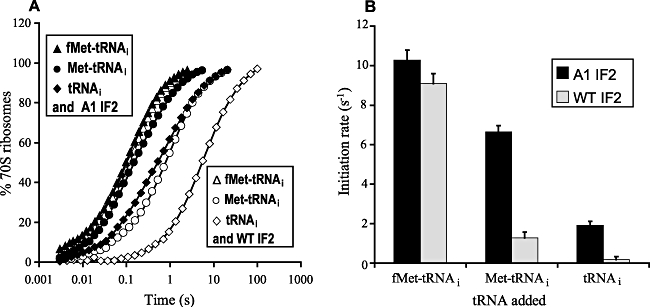
Effect of tRNA methionylation and subsequent formylation on the rate of 50S docking to tRNA-containing 30S PICs assembled with WT IF2 or A1 IF2 mutant. A. 50S subunits were rapidly mixed with 30S PICs assembled with WT IF2 or A1 IF2 mutant and 2 µM deacylated tRNA_i_, Met-tRNA_i_ or fMet-tRNA_i_. B. Rates (*k*_I_) of 70S formation for the experiments in (A).

We studied the effects of tRNA acylation and formylation on the rate of 50S docking to the complete 30S PIC by rapidly mixing 50S subunits with 30S PICs already containing either fMet-tRNA_i_, Met-tRNA_i_ or deacylated tRNA_i_ (Fig. 8). In this experiment, with the tRNA binding step omitted, removal of the formyl group of fMet-tRNA_i_ led to a sevenfold reduction in subunit joining rate (from 9 to 1.3 s^−1^) with wild-type IF2, whereas for the A1 mutant the rate reduction was less than twofold (from 10 to 6.6 s^−1^). Removal of methionine led to a further sevenfold reduction in the subunit joining rate (from 1.3 to 0.18 s^−1^) for wild-type IF2 and a threefold reduction for the A1 mutant IF2 (from 6.6 to 2.2 s^−1^). These observations show that the loss of formylation and methionylation of fMet-tRNA_i_ had a much smaller effect on subunit joining with the A1 IF2 mutant than with wild-type IF2 (5- versus 50-fold reduction in the subunit joining rate).

With regard to wild-type IF2, the sevenfold reduction in subunit joining rate due to removal of the formyl group of fMet-tRNA_i_ observed here (Fig. 8) is much smaller than the previously reported 50-fold rate reduction (Antoun *et al.*, 2006b). Here we have observed such a reduction only with deacylated tRNA_i_. This suggests that the reason for the discrepancy between the present and the previous results could be a fast de-acylation of pre-charged purified Met-tRNA_i_ used in previous experiments (Antoun *et al.*, 2006b). In all experiments in this study the methionylation level of Met-tRNA_i_ was kept high by the presence of Met, MetRS and ATP in the reaction mixtures.

## Discussion

Disruption of the *fmt* gene in eubacteria results in a four- to 10-fold reduction in growth rate due to inefficient initiation of protein synthesis with non-formylated initiator tRNA (Guillon *et al.*, 1992; 1996; Steiner-Mosonyi *et al.*, 2004; Nilsson *et al.*, 2006). This formylation deficiency can, however, be compensated by several types of mechanisms, including an increase in Met-tRNA_i_ concentration by amplification of the *metZ* and *metW* genes, encoding tRNA_i_ (Nilsson *et al.*, 2006), an increase in IF2 concentration (Guillon *et al.*, 1996) or an increase in IF2 affinity to Met-tRNA_i_ by mutations in the initiator tRNA binding domain IV of IF2 (Steiner-Mosonyi *et al.*, 2004). Here, we isolated several fast-growing mutants with amino acid substitutions in IF2 that compensated for the lack of the FMT enzyme. These different mutations increased the growth rate by two- to threefold relative to that observed with wild-type IF2. Differently to what was observed for previously isolated compensatory mutations in IF2 (Steiner-Mosonyi *et al.*, 2004), none of the mutations found here were located in the fMet-tRNA_i_ binding domain IV of IF2, which excluded any direct effect of these mutations on IF2 affinity to Met-tRNA_i_. Instead, most mutations (8 of 13) were found in domain III of IF2, two in the linker region of IF2 connecting domains III and IV, two in the G domain and one in the N-terminal domain of IF2 (Table 1, Fig. 2). The mutations could be roughly separated into two classes (A and B) according to the extent of their compensatory effect on the growth rate in the *fmt* background (Fig. 1). Importantly, we found that the fitness cost in formylation-proficient strains was most severe for the class A mutations in IF2 that showed the strongest compensatory effect (Fig. 3).

### Effects of IF2 mutations on *in vitro* initiation account for their *in vivo* phenotypes

During initiation of bacterial protein synthesis, IF2 plays pivotal roles in the binding of fMet-tRNA_i_ to the 30S pre-initiation complex and in the subsequent fast docking of the 50S subunit to the 30S PIC containing fMet-tRNA_i_ (Benne *et al.*, 1973; Wintermeyer and Gualerzi, 1983; Antoun *et al.*, 2003; Antoun *et al.*, 2006a; Grigoriadou *et al.*, 2007). Biochemically, these two steps were jointly studied here in experiments where 50S subunits and fMet-tRNA_i_ or Met-tRNA_i_ were rapidly mixed with tRNA_i_ lacking 30S PICs in a stopped-flow instrument to monitor 70S initiation complex formation by light scattering (Antoun *et al.*, 2004).

Initiation with fMet-tRNA_i_ occurred with similar rate, *k*_I_, for all mutant and wild-type IF2s (Fig. 4). The rate varied little with the fMet-tRNA_i_ concentration (Fig. 4D), suggesting that subunit docking and not tRNA_i_ binding was the rate limiting step, in line with previous results with wild-type IF2 (Antoun *et al.*, 2006a). However, initiation with Met-tRNA_i_ occurred with much lower, and Met-tRNA_i_ concentration-sensitive, *k*_I_-values for the A- and B-mutant IF2 classes and wild-type IF2 (Fig. 5, Table 2). Such a strong dependence of the *in vitro* initiation rate on Met-tRNA_i_ concentration also for wild-type IF2 (Fig. 5) explains why the amplification of initiator tRNA genes has a strong compensatory effect on the growth rate of *fmt*-deficient strains harbouring wild-type IF2 (Nilsson *et al.*, 2006).

The initiation rate, *k*_I_, approached *k*_max_*-*values of 1, 2 and 4 s^−1^ for wild-type, class B and class A IF2 mutants, respectively, at saturating concentration of Met-tRNA_i_ (Table 2), showing that the subunit joining rate in the presence of non-formylated Met-tRNA_i_ was twofold faster for class B and fourfold faster for class A IF2 mutants than for wild-type IF2. In addition, there was a strong linear correlation between the *in vitro* initiation time, 1/*k*_I_, with Met-tRNA_i_ and the generation time of the formylation-deficient strains harbouring the corresponding mutant and wild-type IF2s (Fig. 6). The linear correlation also included the initiation and generation times for wild-type IF2 with fMet-tRNA_i_. Thus, the initiation time measured *in vitro* and the cell generation time displayed a linear dependence in a broad range of generation times (Fig. 6), and we concluded that the enhanced initiation efficiency with Met-tRNA_i_ for the IF2 mutants as compared with wild-type IF2 accounts for the growth compensatory effects of these mutations under formylation-deficient conditions.

### Fitness cost and formation of aberrant 70S complexes with class A IF2 mutants

*In vitro* initiation with fMet-tRNA_i_ was faster with the class A IF2 mutants than with wild-type IF2 (Fig. 4F). In contrast, formylation-proficient strains with class A mutations in IF2 grew more slowly than wild-type strain (Fig. 3). Our *in vitro* experiments suggest that the explanation for the reduction in growth rate of the class A IF2 mutants is an increased frequency of aberrant initiation events.

*In vitro* initiation with deacylated tRNA_i_ and class A or class B IF2 mutants was about fivefold or twofold faster, respectively, than initiation with wild-type IF2 (Fig. 7B). Furthermore, *in vitro* initiation with the acylated elongator Phe-tRNA^Phe^ was about fivefold faster with the A1 mutant than with the wild-type IF2 (Fig. 7D). Since the initiation rates with wild-type and mutant IF2s were similar in the presence of fMet-tRNA^fMet^ (Fig. 4F), these results suggest a higher frequency of aberrant initiation events in the strains harbouring class A IF2 mutants than in IF2 wild-type strain. Aberrant 70S complexes containing deacylated tRNA can be efficiently recycled back to the 30S and 50S subunits by the joint action of ribosomal recycling factor RRF and elongation factor EF-G (Pavlov *et al.*, 2008). In contrast, aberrant 70S complexes with acylated elongator tRNAs are resistant to recycling and will participate in protein elongation, leading to out-of-frame initiation and the appearance of erroneous and potentially harmful intracellular proteins (Hirokawa *et al.*, 2004; Pavlov *et al.*, 2008). Elevated levels of aberrant initiation events with acylated elongator tRNAs in the formylation-proficient background in bacteria harbouring class A IF2 mutations may therefore be the primary reason for the significant fitness reduction associated with these mutants (Fig. 3).

### The conformation of mutant IF2s on the 30S subunit

The rate of subunit docking with fMet-tRNA_i_ in the 30S PIC was similar for wild-type and A1 mutant IF2 (Fig. 8). When, however, deacylated tRNA_i_ replaced fMet-tRNA_i_, this rate dropped down 50-fold for wild-type IF2 but only fivefold for the A1 mutant IF2 (Fig. 8). Also, in the absence of any tRNA in the P site of the 30S PIC, the slow subunit docking was noticeably more efficient with the A1 mutant than with wild-type IF2 (Fig. 4A). Accordingly, the A1 mutation in IF2 made the rate of docking of the 50S subunit to the pre-initiation 30S complex much less dependent on formylation/methionylation of tRNA_i_ or even the presence of the initiator tRNA. Thus, we speculate that the class A mutations in the domain III of IF2 led to a higher propensity of 30S bound IF2 to acquire its 50S docking conformation (Antoun *et al.*, 2006a; Simonetti *et al.*, 2008) even in the absence of tRNA. Addition of deacylated tRNA_i_ or Met-tRNA_i_ led to successively higher stabilization of the 50S docking conformation for mutant and wild-type IF2, with the former at an advantage due to its intrinsic propensity for the docking conformation. In the presence of the authentic initiator tRNA, fMet-tRNA_i_, both A1 mutant and wild-type IF2 had almost completely switched conformation to the docking prone form, and hence the rate of subunit docking with fMet-tRNA_i_ was much more similar for these two IF2 variants (Fig. 8).

### Evidence for mRNA-limited protein synthesis

The initiation times (equal to the inverse of the effective initiation rates, 1/*k*_I_), estimated from our biochemical experiments for the different IF2 variants (Fig. 5F), correlate linearly with the generation times (equal to the inverse of the growth rates) of the corresponding bacterial strains (Fig. 6). The shortest initiation time (0.25 s), obtained for fMet-tRNA_i_ and wild-type IF2 (Fig. 4F), corresponds to the shortest generation time (25.2 min) of a formylation-proficient strain with wild-type IF2. The longest initiation time (2.8 s), obtained for Met-tRNA_i_ and wild-type IF2, corresponds to the longest generation time (101 min) of a formylation-deficient strain with wild-type IF2. How can a 2.5 s increase in initiation time result in a fourfold increase in generation time (Fig. 6)? The explanation, we propose, is mRNA-limited protein synthesis. This is a physiological state in which the total rate of protein synthesis per cell volume, *V*_p_, is insensitive to the total ribosome concentration and proportional to the total mRNA concentration, [*mRNA*_0_], in the cell. This rate is given by *V*_p_ = [*mRNA*_0_] × *n*_c_/*τ*_reg_ (see *Experimental procedures* for details), where *n*_c_ is the average number of codons per mRNA and *τ*_reg_ is the time after 30S subunit binding to the mRNA for 70S initiation complex formation (*τ*_70S_) plus the time (*τ*_clear_) for ribosomal translation in the open reading frame far enough to allow for the binding the next 30S subunit to the same mRNA. Since *V*_p_ is proportional to the bacterial growth rate (Ehrenberg and Kurland, 1984), the generation time is directly proportional to *τ*_reg_ = *τ*_70S_ + *τ*_clear_ under mRNA-limited conditions.

The shortest possible distance between ribosomes observed in tightly packed bacterial polysomes is about 72 nucleotides (nts) (Brandt *et al.*, 2009) which, at an elongation rate of 20 aminoacids per second (60 nt s^−1^), corresponds to *τ*_clear_ = 1.2 s (= 72 nts/60 nt s^−1^). Our biochemical experiments show that the time, *τ*_70S_, for formation of a 70S initiation complex with fMet-tRNA_i_ and wild-type IF2 was 0.25 s. Adding these times, one obtains a minimal time, *τ*_reg_, of 1.45 s between initiation events on mRNA in a wild-type cell with a generation time of 25 min, corresponding to an average ribosome-to-ribosome distance, D, on mRNA of 87 nts (obtained as *D* = 1.45 s × 60 nt s^−1^). In the case of non-formylated Met-tRNA_i_ and wild-type IF2, 70S initiation complex formation proceeded slowly with *τ*_70S_ = 2.8 s (Fig. 6). In this case *τ*_reg_ = *τ*_70S_ + *τ*_clear_ = 2.8 s + 1.2 s = 4 s, which corresponds to an expected ribosome-to-ribosome distance on mRNA *in vivo* of 240 nts. The approximately threefold increase in *τ*_reg_ (4 s/1.45 s) due to the lack of Met-tRNA formylation is close to the fourfold increase in generation time observed *in vivo* (Fig. 6). Furthermore, a greatly reduced ribosome density in polysomes (by a factor of 240/87) may have increased the accessibility of mRNA to ribonucleases and, hence, increased the bulk mRNA degradation rate (Arnold *et al.*, 1998; Regnier and Arraiano, 2000; Sunohara *et al.*, 2004), amplifying the primary effect of prolonged initiation times on *in vivo* generation time. Thus, under the assumption that bulk protein synthesis is mRNA-limited, our biochemical data account for the generation times measured *in vivo*.

The weight fraction of mRNA per total RNA at moderate growth rates was estimated to be about 2.5%, while the fraction of ribosomal RNA (rRNA) plus transfer RNA (tRNA) was about 98% (Baracchini and Bremer, 1987). From these numbers a ribosome-to-ribosome distance, D, in polysomes was estimated as 115 nts at a generation time (*τ*_g_) of 45 min (Baracchini and Bremer, 1987). More refined estimates for this distance at fast growth rates obtained recently by a similar method (Bremer and Dennis, 2008) show that *D* = 88 nts at *τ*_g_ = 24 min and *D* = 69 nts at *τ*_g_ = 20 min. The *D*-value of 88 nts corresponds well with our model estimate *D* = 87 nts for *τ*_g_ = 25 min (see above). Importantly, the distance of 69 nts is below the *D*-limit of 72 nts for the tightest ribosome packing in polysomes (Brandt *et al.*, 2009), indicating that at the highest growth rate there is not enough mRNA in the cell to accommodate all ribosomes and protein synthesis is therefore mRNA-limited. This implies that in our experiments the wild-type strain was growing at or near mRNA-limited conditions, while the various mutants were growing under strict mRNA-limited conditions. From this interpretation follows the prediction that the formylation-deficient strains with impaired initiation had larger fractions of free 70S ribosomes and ribosomal subunits than the wild-type formylation-proficient strain, as previously found for strains with reduced IF2 concentration (Cole *et al.*, 1987).

### Development of antibiotic resistance

Finally, the presented data are highly relevant for the question of antibiotic resistance development. Antibiotic resistance is usually associated with reduced fitness (Andersson and Levin, 1999; Andersson, 2006) and at a given antibiotic pressure this fitness cost is a main determinant of the rate of development as well as the steady-state level of resistance (Levin *et al.*, 1997; Levin, 2002). The association of resistance with decreased fitness suggests that a reduction in the use of antibiotics would lead to a reduction in the frequency of resistant bacteria by natural selection. However, the fitness cost of resistance can be reduced at unaltered resistance by additional second-site compensatory mutations (Andersson and Levin, 1999; Andersson, 2006). The fitness costs associated with *fmt* mutations that cause resistance to PDFIs can be reduced by increased tRNA_i_ expression via gene amplification of the *metZW* genes (Nilsson *et al.*, 2006) or by point mutations in IF2 (this work). This multitude of different compensatory pathways suggests that the fitness costs of *fmt* mutations conferring resistance to PDFIs can be rapidly reduced by mutations, increasing both the rate of development and the steady-state level of PDFI resistance in clinical settings. A deeper understanding of the mechanisms by which bacteria reduce the fitness cost associated with drug resistance helps in the choice and development of drugs and drug targets for which adaptation is slow.

## Experimental procedures

### Strains

For all experiments, except where specifically indicated, the organism used was *S. enterica* serovar *typhimurium* LT2 (*S. typhimurium*). The minimal inhibitory concentrations (MICs) for the wild-type strain, the actinonin resistant *fmt* mutants and the *fmt*, *infB* double mutants were, 64 mg l^−1^, > 1024 mg l^−1^ and > 1024 mg l^−1^ respectively.

### Compensatory evolution and identification of compensatory mutations

Two slow-growing actinonin-resistant mutants with mutations in *fmt* were subjected to compensatory evolution. For each strain, 10–15 independent lineages were serially passaged in Luria–Bertani (LB) broth. When growth-compensated cells constituted the majority of the population (50–150 generations of growth), one compensated mutant clone from each lineage was isolated and saved at −80°C. To identify the unknown compensatory mutations the mini-Tn*10* transposon insertion technique was used (see Supporting information).

### Hydroxylamine mutagenesis

To isolate additional IF2 mutants, hydroxylamine mutagenesis of DNA inside bacteriophage P22 was performed. To this end we used the starting strain (DA2964), which has an *argG1828*::Tn10 (Tet^R^) marker linked to the *infB* gene. A high-titre (7.5 × 10^12^ pfu ml^−1^) phage lysate (P22 HT) was prepared on this strain and the DNA inside the phage was mutagenized as described previously (Hong and Ames, 1971).

The resulting mutagenized phage lysate was used to transduce a Δfmt mutant strain (DA10066) by mixing 5 µl mutagenized phage lysate with 200 µl of an overnight bacterial culture and incubating for 1 h at 37°C. The mixture was then plated on LA supplemented with 30 mg l^−1^ tetracycline and the plates were incubated for 48 h at 37°C. Fast growers were picked, re-streaked on tetracycline and purified on EBU plates. The picked clones were then confirmed to be P22 sensitive and phage free. Nine individual fast growers were isolated and their *infB* gene sequenced.

### Fitness of mutants

Fitness of the *infB* mutants was measured in both Δ*fmt* and wild-type genetic backgrounds. Using a phage lysate grown on strain DA2964, an *argG*1828::Tn*10* insertion genetically linked to *infB* was transduced into the different *infB* mutant strains. Subsequently, the different *infB* mutants were introduced by P22 transduction into strains DA6192 (wild-type) and DA10066 (*Δfmt*). Presence of the correct *infB* mutations was confirmed by sequencing.

### Complementation study

For complementation studies we prepared pBAD30::mut_infBHIS plasmids for each mutant IF2 as described in Supporting Information. The same plasmids were also used for over-production of IF2 mutants used in the *in vitro* studies. Different pBAD30::mut_infBHIS constructs were transformed by electroporation into the Δ*fmt* mutant (DA10066). Fitness of all complemented mutants was estimated by measuring growth rates in rich medium. The bacteria were pre-grown to saturation in LB overnight. Approximately 10^6^ cells were inoculated into 0.4 ml of fresh LB in a Bioscreen plate and the absorbance at 600 nm was read with a BioscreenC (Oy Growth Curves Ab Ltd). For each strain, growth rates were measured in quadruplicates in at least two separate experiments and relative growth rates were calculated as the growth rate of the parental strain divided by the growth rate of the tested strain.

### Chemicals and buffers for *in vitro* experiments

Phosphoeno*l*pyruvate (PEP), myokinase (MK), pyruvate kinase (PK), inorganic pyrophosphotase (PPi), putrescine and spermidine were from Sigma (USA). Experiments were conducted in a polymix-like buffer, LS4, containing 95 mM KCl, 3 mM NH_4_Cl, 0.5 mM CaCl_2_, 8 mM putrescine, 1 mM spermidine, 30 mM HEPES pH 7.5, 1 mM DTE, 2 mM PEP, 1 mM GTP, 1 mM ATP and 5 mM Mg(OAc)_2_, supplemented with 1 µg ml^−1^ PK and 0.1 µg ml^−1^ MK (Jelenc and Kurland, 1979; Pavlov *et al.*, 2008). Since each ATP or GTP molecule chelates one Mg^2+^ cation, the free Mg^2+^ concentration in the LS4 buffer was adjusted to 4 mM by adding 1 mM Mg(OAc)_2_.

### Components of the *in vitro* translation system

70S ribosomes, 50S and 30S subunits, [^3^H]fMet-tRNA_i_, initiation factors as well as Met and Phe animacyl-tRNA synthetases (MetRS and PheRS) were prepared from *Escherichia coli* as described in Freistroffer *et al.* (1997), Antoun *et al.* (2004) and Antoun *et al.* (2006a). Overproduced N-terminus-His-tagged wild-type and mutant *S*. *typhimurium* IF2s were isolated from *S. typhimurium* essentially as described in Antoun *et al.* (2004). Initiation tRNA_i_ and tRNA^Phe^ were from Sigma (USA). mMFTI mRNA and mFMTI mRNA with strong SD sequences were prepared as described in Pavlov *et al.* (2008).

Comparison of the amino acid sequences of wild-type IF2 from *S. typhimurium* and *E. coli* showed that they were > 96% identical. The identity level was even higher, > 98%, when the functionally dispensable N-terminal domain of IF2 (Caserta *et al.*, 2006) was excluded. Accordingly, wild-type IF2s from *S. typhimurium* and *E. coli* behaved practically identically in all *in vitro* initiation experiments (data not shown), which justifies the use of the well-characterized *E. coli* components in the biochemical experiments conducted in this study.

### *In vitro* kinetic experiments

Two mixtures, 1 and 2, were prepared in the LS buffer. Mixture 1 contained 30S pre-initiation complexes assembled by mixing 0.32 µM 30S subunits, 0.8 µM mMFTI mRNA with a strong SD sequence (Pavlov *et al.*, 2008), 1 µM initiation factor IF1, 0.6 µM IF2 and 0.5 µM IF3 unless specified otherwise. Mixture 2 contained 0.36 µM 50S subunits. The type and final concentration of tRNA added either to mixture 1 or mixture 2 is indicated for each experiment in the corresponding figure legend. Both mixtures (1 and 2) were pre-incubated for 20 min at 37°C. Met-tRNA_i_ was methionylated *in situ* as follows. First, a mixture containing 200 µM initiator tRNA, 1 mM [^3^H]Met aminoacid and 800 unit ml^−1^ MetRS in LS4 buffer supplemented with 1 µg ml^−1^ PK, 0.1 µg ml^−1^ PPi and 0.1 µg ml^−1^ MK (Jelenc and Kurland, 1979) was assembled and pre-incubated for 20 min at 37°C after which it was put on ice. Just before loading into a stopped flow instrument (see below), a portion of this mixture was added to mixture 2 containing 50S subunits (or to mixture 1 containing 30S subunits) to obtain the final Met-tRNA_i_ concentration specified for each experiment. Phe-tRNA^Phe^ was prepared in the same way except that the acylation mixture was assembled with tRNA^Phe^, Phe aminoacid and PheRS instead of tRNA_i_, Met amino acid and MetRS. Pre-incubated mixtures 1 and 2 (0.6–0.8 ml volume of each) were loaded into the syringes of a stopped flow instrument (SX-20, Applied Photophysics, Leatherhead, UK). The kinetics of 70S complex formation was monitored at 37°C with light scattering after rapid mixing equal volumes (usually 0.06 ml) of mixtures 1 and 2 as described (Antoun *et al.*, 2006a). All concentrations in mixes 1 and 2 specified above are the final concentrations after the mixing.

### Treatment of light scattering data

Each light scattering experiment provided 6–8 scattering traces. Those were used to obtain an average scattering trace and to estimate the average and standard deviation of the time, *t*_0.5_, at which a light scattering trace reached 50% of its plateau value. The time *t*_0.5_ obtained for the average trace was always very close to the average *t*_0.5_ time. The rate, *k*_I_, of the initiation reaction was defined as the inverse of *t*_0.5_ for the average trace as motivated by the following relation between scattering intensity and time:


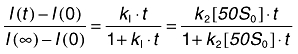
(1)

This relation describes the irreversible formation of a binary complex, A:B, from particles A and B mixed at equal concentrations (Antoun *et al.*, 2006a). The rate *k*_I_ in this relation is equal to the product of the second-order rate constant *k*_2_ of the binding reaction by the initial concentration of B particles (50S subunits in our case). In the presence of IF3 expression (1) holds, but *k*_2_ is now a compounded rate constant that depends on the concentrations of both IF3 and 50S subunits (Antoun *et al.*, 2006a).

When the initiation reaction includes tRNA binding to the 30S PIC with association rate constant *k*_1_ followed by the 50S subunit docking with rate constant *k*_2_, then *t*_0.5_ is approximated by (Supporting information):


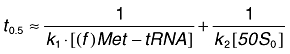
(2)

The L-B representation of this expression is:


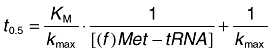
(3)

Comparison of (2) and (3) clarifies the relation between rate constants *k*_1_ and *k*_2_ and L-B parameters *k*_max_/*K*_M_ and *k*_max_.

The scattering traces were fitted to a four-parameter kinetic model (Fig. S4B) describing tRNA binding to active 30S PICs (rate constant *k*_1_), subsequent docking of 50S subunits to tRNA-containing, active 30S PICs (rate constant *k*_2_) and a slow conversion (described by rate constant *k*_3_ and *q*_3_) of a small fraction of subunit-docking-inactive 30S PICs (see Supporting information for details). For clarity of presentation we used a digital filter to reduce the noise and the numbers of data points in the scattering traces shown in the figures.

### Definition of mRNA-limited protein synthesis in growing cells

The total rate of protein synthesis per cell volume can be written as (see section D in Supporting information for details):


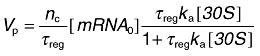
(4)

Here, [*mRNA*_0_] is the total mRNA concentration in the cell, *n*_c_ is the average number of codons per mRNA, [*30S*] is the concentration of free 30S subunits and *k*_a_ is the rate constant for 30S subunit association to mRNA. The time *τ*_reg_ = *τ*_70S_ + *τ*_clear_ is the time after 30S subunit binding to the mRNA for 70S initiation complex formation (*τ*_70S_) plus the time (*τ*_clear_) for ribosomal translation of the mRNA ORF far enough to allow for the binding of a next 30S subunit to the mRNA. The quadratic equation:


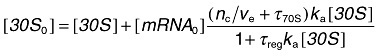
(5)

relates [*30S*] to the total concentration, [30*S*_0_], of the 30S subunit (equal to the total ribosome concentration) and [*mRNA*_0_]. Here, *v*_e_ is the elongation rate of translating ribosome in codons per second. Together, relations (4) and (5) determine how *V*_p_ depends on [*mRNA*_0_] and [30*S*_0_] for any choice of the parameters *τ*_70S_, *τ*_clear_, *k*_a_, *n*_c_, *v*_e_, as exemplified in Fig. S5.

In the limiting case, where *τ*_reg_*k*_a_[*30S*] >> 1, relation (4) is approximated by:


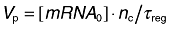
(6)

This defines the condition of mRNA limitation, where *V*_p_ is proportional to [*mRNA*_0_] and inversely proportional to *τ*_reg_. In this limit, relation (5) is approximated by:


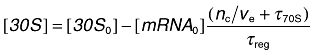
(7)

The ratio 
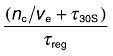
 in relation (7) defines the maximal number of ribosomes on an mRNA with *n*_c_ codons (see section D of the Supporting information). From relation (7) follows that the condition *τ*_reg_*k*_a_[*30S*] >> 1 for mRNA-limited protein synthesis requires that


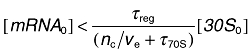
(8)

When *τ*_reg_*k*_a_[30*S*_0_] >> 1, as in the realistic cases illustrated in Fig. S5, inequality (8) approximates the region in which protein synthesis is mRNA-limited.
