# Supplementary material for: Error-prone initiation factor 2 mutations reduce the fitness cost of antibiotic resistance
Source: Mol Microbiol. 2010 Feb 3;75(5):1299–313. doi: 10.1111/j.1365-2958.2010.07057.x (PMC2859245; doi:10.1111/j.1365-2958.2010.07057.x)
Supplement: Supplementary file 1 [file mmi0075-1299-SD1.pdf]

Supporting Information

Supplementary Figures

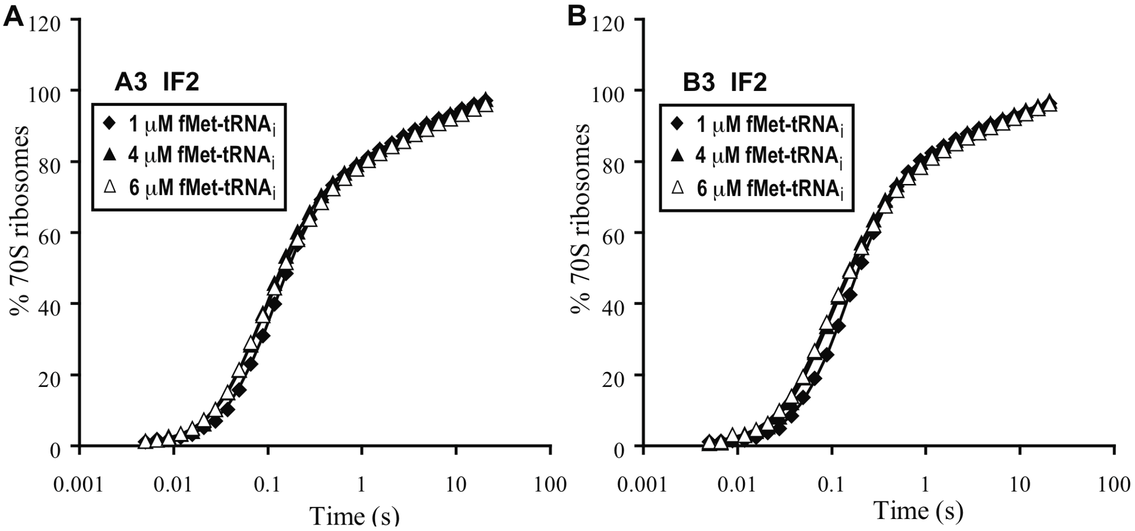

Figure S1. Kinetics of 70S initiation complex formation after mixing tRNA-free 30S PICs with 50S subunits and fMet-tRNA$_i$ and their dependence on fMet-tRNA$_i$ concentration. **(A)** 30S PICs containing A3 IF2 were rapidly mixed with the mixture containing 50S subunits and fMet-tRNA$_i$ in 1, 4 or 6 μM concentration. **(B)** The same as **(A)** but 30S PICs contained B3 IF2. All concentrations in the figures are the final concentrations after the mixing.

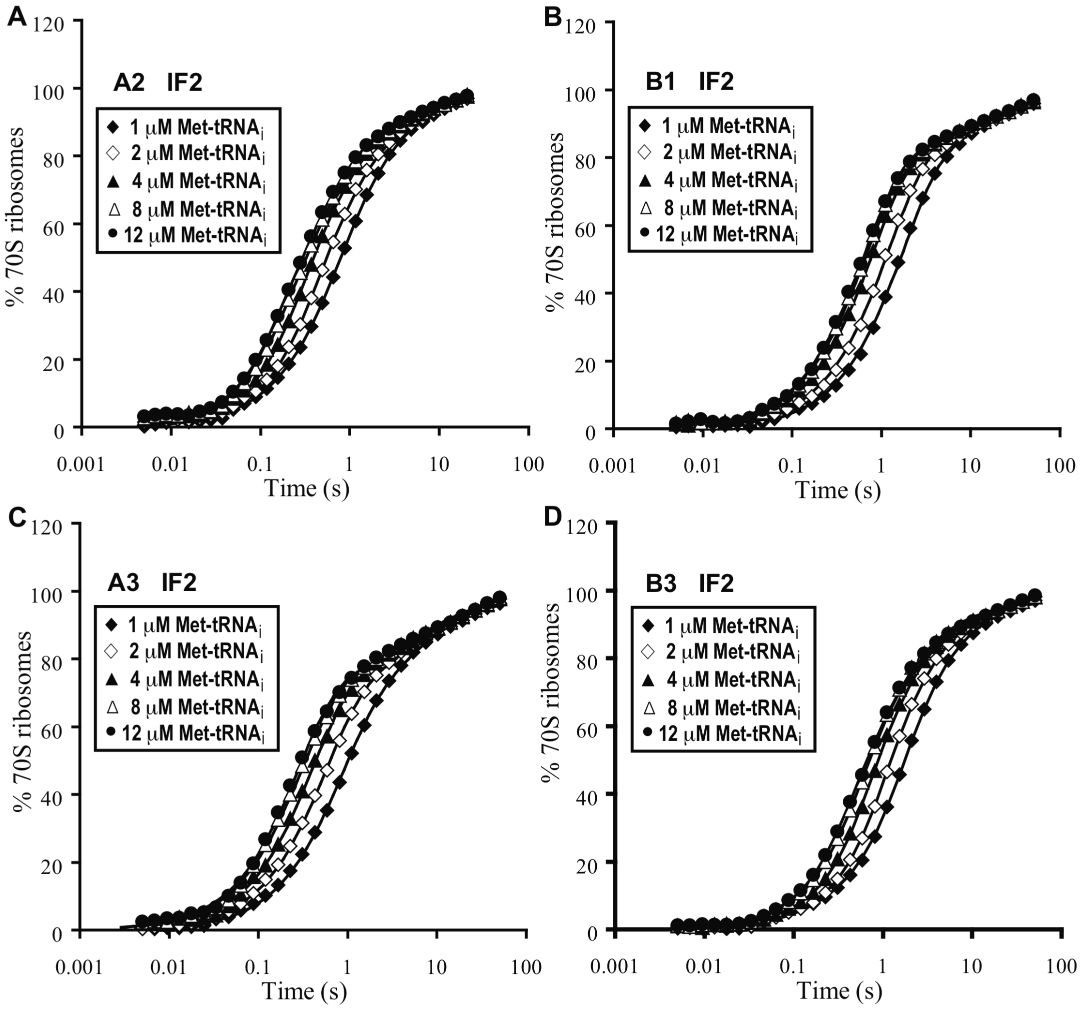

Figure S2. Kinetics of 70S initiation complex formation after mixing tRNA-free 30S PICs with 50S subunits and unformylated Met-tRNA$_i$ and their dependence on Met-tRNA$_i$ concentration. **(A)** 30S PICs containing A2 IF2 were rapidly mixed with the mixture containing 50S subunits and Met-tRNA$_i$ in 1, 2, 4, 8 or 12 μM concentration. **(B)** The same as **(A)** but 30S PICs contained B1 IF2. **(C)** The same as **(A)** but 30S PICs contained A3 IF2. **(D)** The same as **(A)** but 30S PICs contained B3 IF2. All concentrations in the figures are the final concentrations after the mixing.

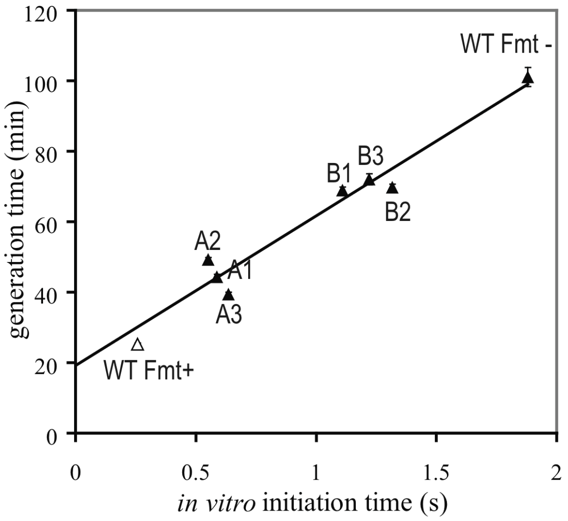

Figure S3. Correlation between generation times of *fmt* mutant strains harboring different IF2s and the *in vitro* initiation times measured with 2 μM Met-tRNA$_i$ added together with 50S subunits to tRNA-free 30S PIC containing corresponding IF2s.

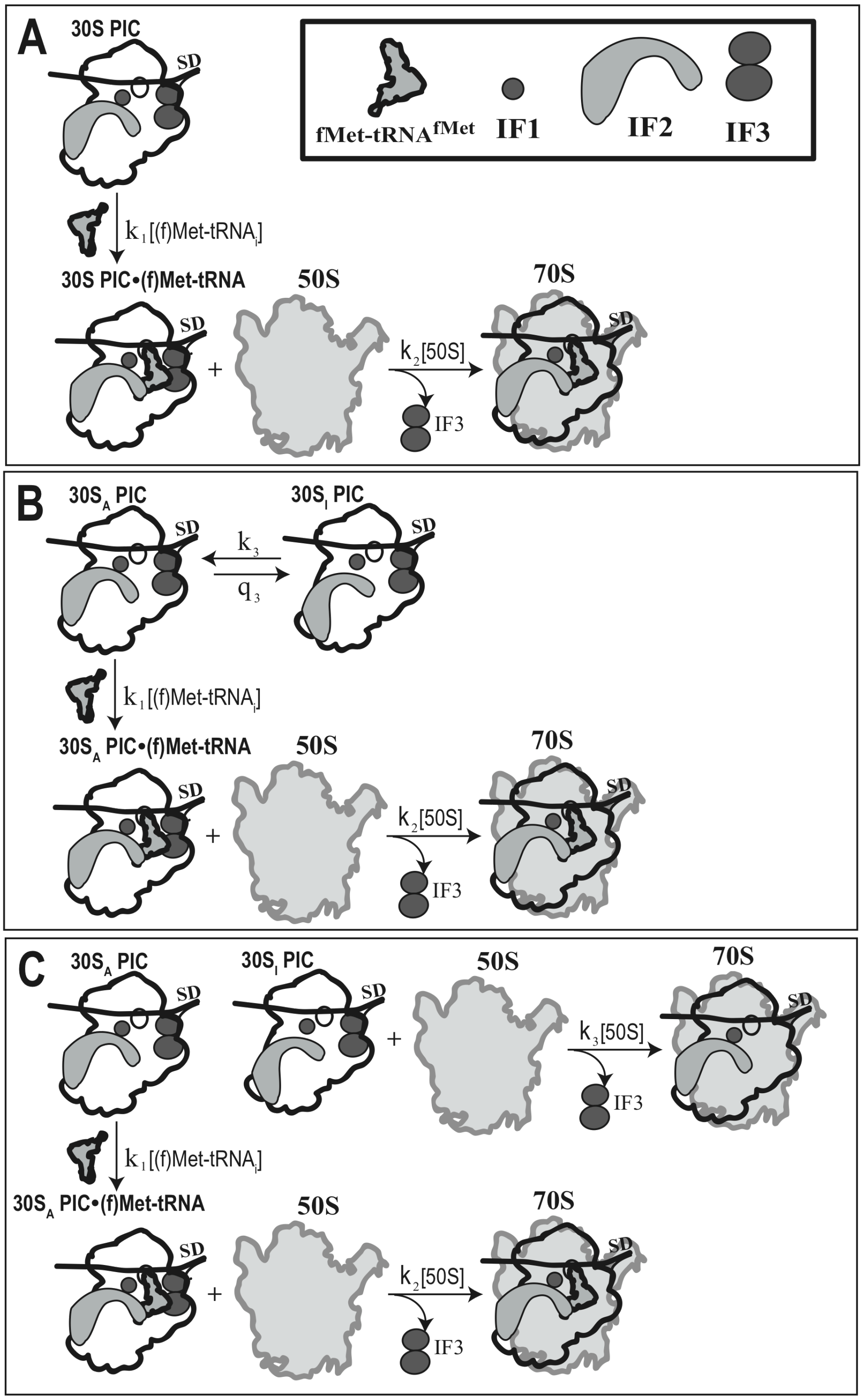

Figure S4. Kinetic model of 70S initiation complex formation upon addition of 50S subunits (50S) together with formylated/unformylated (f)Met-tRNA to the 30S PICs (30S). **(A)** 30S PIC binds (f)Met-tRNA with rate constant k_1; the tRNA containing 30S PIC binds then to the 50S subunit with the rate constant k_2. **(B)** The same as in **(A)** but 30S PICs can be either in active $30S_A$ PIC or inactive $30S_I$ PIC conformation and the transition between them occurs with rate constants k_3 and q_3. **(C)** The same as in **(A)** but inactive $30S_I$ PICs do not transform into the active complexes but, instead, can bind 50S subunits slowly with second order rate constant k_3. Approximate positions of initiation factors IF1, IF2, IF3 as well as the site of SD:anti-SD interactions (SD) on the 30S subunit are shown.

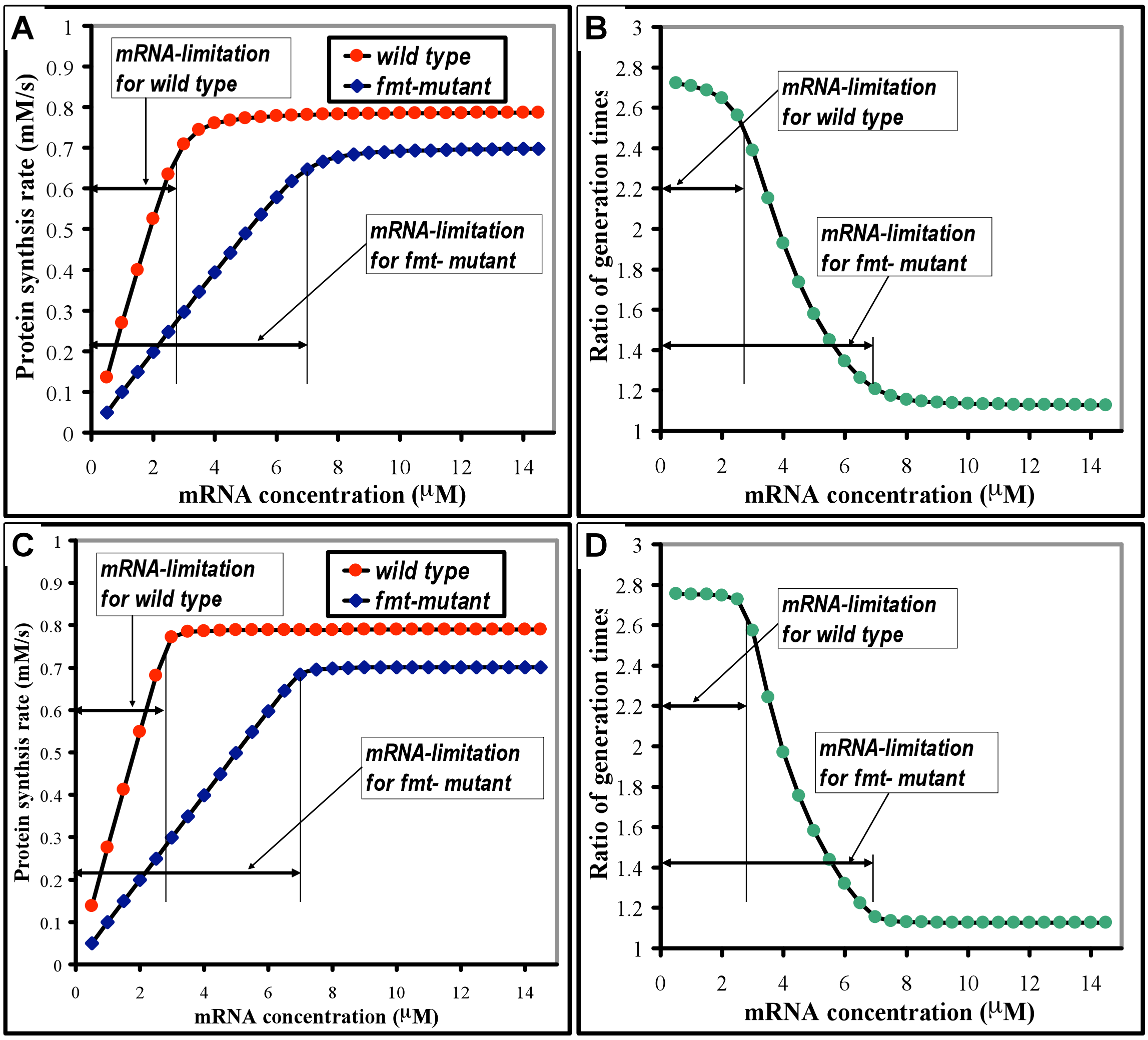

Figure S5. Dependence of the total rate of protein synthesis on mRNA concentration in the cell. (A) Total rate of protein synthesis V_p (in mM/s) was calculated for different mRNA concentrations according to relations (D12) and (D14) using the following values of parameters: $[30S_0]$=40 μM;, $\tau_{clea,}$=1.2 s; n_c=1200 nts;, v_e=20 aa/s (60 nts/s) ; k_a=1 $\mu M^{-1}s^{-1}$; τ_{70S}=0.25 s for wild type IF2 and fMet-tRNA$_i$ and τ_{70S}=2.8 s for wild type IF2 and non-formylated Met-tRNA$_i$. (B) Ratio of generation times for bacterial strains having formylation deficient and formylation proficient genetic background harboring wild type IF2 calculated as the ratio of the corresponding rates of protein synthesis in (A). (C) The same as in (A) but k_a =10 $\mu M^{-1}s^{-1}$ was used in V_p calculations, instead of k_a =1 $\mu M^{-1}s^{-1}$. (D) The same as in (B) but the rates of protein synthesis in (C) were used to calculate the ratio of generation times. Regions where the rate of total protein synthesis in the cell is mRNA-limited according relation (D17) are indicated for the wild type and *fmt-* mutant strains in the figure.

Supplementary Materials and Methods

A. Identification of unknown compensatory mutations using the mini-Tn10 transposon insertion technique.

Transposon pools with random insertions of the mini transposon Tn*10*Δ16Δ17 [mini-Tn*10*, (Tet^R)] were prepared in two growth compensated mutant strains (DA8740 and DA8799) as described previously (Altman et al., 1996). Each pool consisted of at least 10,000 independent transposon hops. P22 phage was grown on each pool and the phage lysate was used to infect the slow growing parental strain (DA8326 and DA8340, respectively). Tetracycline resistant colonies were selected on LA plates supplemented with 30mg/L tetracycline and the transductants were screened for acquisition of the growth compensated phenotype by visual examination of colony size. Linkage between the growth compensated phenotype and the tetracycline resistant marker was established by back crossing to the slow-growing mutant. An isogenic pair of tetracycline resistant, slow-growing and tetracyclin resistant, fast-growing strains was saved and frozen at -80°C.

Arbitrary primed PCRs directed outwards from the mini-Tn*10* transposon was performed to identify the insertion points of the transposons. The PCR was performed in two steps. First, a PCR reaction was set up with one specific primer for the transposon and a mix of arbitrary primers. The arbitrary primers used consisted of a defined part (20 bp) and a variable part (15 bp) where bases are inserted randomly. Using the PCR products from the first reaction as template, a second nested PCR was performed. The product from the second reaction PCR reaction was then used as a template for sequencing.

For the transposon linked to the compensatory mutation in DA8740, the insertion point was the *dacB* gene. Similarly, for strain DA8799 the insertion point of a transposon linked to the compensatory mutation was the STM3278 open reading frame. The *dacB* gene and open reading frame STM3278 are located in the same region on the Salmonella chromosome, indicating that the target for compensatory mutations in both DA8740 and DA8799 might be identical. Located between these two genes is the *infB* gene, encoding initiation factor 2 (IF2) (8,884 bp between STM3278 and *infB* and 12,366 bp between *infB* and *dacB*). PCR amplification and sequencing of the *infB* gene revealed point mutations in both growth compensated strains. The isolated transposon strains were subsequently used for linkage experiments to identify additional growth compensated strains with mutations in *infB*. Three more strains were identified that carried point mutations in *infB* and a total of five growth compensated strains with mutations in *infB* were isolated in the serial passage experiments.

B. Construction of pBAD30::mut_infB$_{HIS}$ plasmids for complementation studies and IF2 over-expression.

The pBAD30::mut_infB$_{HIS}$ plasmids were constructed as follows: The mutant *infB* coding region was amplified using PCR with the primer c-infB-forSD-Sac [atatgagctca**aggaga**tatacatatgcac***caccaccaccaccac***acagatgtaaccctaaaagcgc] that contained a *Sac*I restriction site, an optimal **Shine-Dalgarno** sequence, and a ***His-tag*** for purification purposes and the c-infB-rev-xbaI [gcgctctagattaagcgatggtacgttggatct] primer that contained a *Xba*I restriction site. The resulting PCR product was digested with *Sac*I and *Xba*I and ligated into the plasmid pBAD30 pre-digested with the same enzymes (Fermentas) Five µl of the ligation mixture was then transformed by heat shock into chemically competent *E. coli* following the manufacturer's protocol (NEB 5-alpha competent E. coli). Cells were plated on LA supplemented with 100 mg/L ampicillin and incubated overnight at 37°C. Colonies were picked and re-streaked the following day. Plasmid (pBAD30::mut_infB$_{HIS}$) was prepared from one selected clone of each mutant IF2 (E.Z.N.A.® Plasmid Miniprep Kit, Omega Biotech). The plasmid was then transformed into *S. typhimurium* LT2 using electroporation. An overnight culture was diluted 1/100, cells were grown to OD_{600}≈0.5 and expression was induced using 0.2% L-arabinose. (Sigma-Aldrich). After 1.5 hours of induction, cells were pelleted and frozen at -80°C.

C. Kinetics of 70S complex formation in experiments in which tRNA and 50S subunits are added to 30S pre-initiation complexes lacking tRNA.

The kinetic model. The intensity of scattered light after rapid mixing of 30S and 50S subunits supplemented with different factors in a stopped flow instrument reflects the concentration of formed 70S initiation complexes (Antoun et al., 2004). Light scattering data were first fitted to the kinetic scheme in Fig. S4A. Here, the association rate constant k_1 describes tRNA binding to the 30S pre-initiation complex (30S PIC) containing mRNA and all three initiation factors, while the compounded rate constant k_2 describes the subsequent docking of the 50S subunit to the complete tRNA-containing 30S PIC (Antoun et al., 2006). This model did not account for the second, slow phase of 70S formation with small amplitude. We therefore extended the kinetic model in Fig S4A to include two different states, i. e one active and one inactive, of the 30S PIC (Milon et al., 2008). In this model (Fig. S4B) a small fraction of inactive $30S_I$ PICs must become active $30S_A$ PICs with the rate constant k_3 before they bind tRNA and 50S subunits. It is also assumed that before addition of 50S subunits and tRNA the equilibrium between active and inactive conformations of the 30S PIC has already established. The equilibrium fraction of active $30S_A$ PICs determines the amplitude of the major fast phase in light scattering experiments. This fraction is in turn determined by the rate constants k_3 and q_3, so that the model has four parameters k_1, k_2, k_3 and q_3 to be fitted.

Effective rate of 70S initiation complex formation. In the limit of high (f)Met-tRNA$_i$ concentration the product k_1[(f)Met-tRNA$_i$] in the scheme S4B becomes very large in comparison with k_2[50S]. If, in addition, the fraction of inactive 30S PICs is small then a much simpler kinetic scheme emerges:

$$50S + 30S_A \bullet (f)Met - tRNA_i \xrightarrow{k_2 * [50S]} 70S$$

The time course of such a mono-phasic formation of 70S ribosomes is given by the formula (Antoun et al., 2006):

$$[70S](t) = [30S_0] \cdot \left(1 - \frac{\exp(-Qt)}{1 - k_2[30S_0] \cdot (1 - \exp(-Qt))/Q}\right) \qquad \text{(C1)}$$

Here, the apparent rate constant Q depends on the compounded second order rate constant k_2 and the difference between the initial concentrations of the 50S ($[50S_0]$) and 30S ($[30S_0]$) subunits:

$$Q = k_2([50S_0] - [30S_0])$$

When the initial concentrations of 30S and 50S subunits are very close the relation (C1) simplifies to:

$$[70S](t) = [30S_0]\frac{k_2[50S_0]\cdot t}{1+k_2[50S_0]\cdot t} = [30S_0]\frac{k_{2,eff}\cdot t}{1+k_{2,eff}\cdot t} \quad \text{(C2)}$$

Here, $k_{2,eff}$ is the product of the compounded rate constant k_2 and the initial concentration of 50S subunits. The time, $t_{0.5}$, when the concentration of 70S complexes reaches 50% of its maximal value is given by:

$$t_{0.5} = 1/k_{2,eff}$$

Numerical calculations show that for the scheme S4B, $t_{0.5}$ can be approximated by:

$$t_{0.5} \approx \frac{1}{k_1\cdot[(f)Met-tRNA]} + \frac{1}{k_2[50S_0]} = \frac{1}{k_1\cdot[(f)Met-tRNA]} + \frac{1}{k_{2,eff}}$$

provided that the fraction of inactive $30S_I$ PICs is low and the concentration of (f)Met-tRNA$_i$ is sufficiently high to ensure that $k_1\cdot[(f)Met-tRNA] > k_{2,eff}$. This approximation justifies the use of the effective rate k_I, defined as the inverse of $t_{0.5}$, in the main text.

Fitting experimental data to kinetic models. The extended kinetic scheme in Fig. S4B was employed to describe the kinetics of 70S formation when Met-tRNA$_i$ was added together with 50S subunits to the 30S PICs (Fig. 5 and Fig. S2).

Table 1S. Kinetic parameters for *in vitro* initiation with Met-tRNA$_i$ for different IF2s.

	SLWT	B1	B2	B3	A1	A2	A3
$f30S_A$	0.80±0.05	0.81±0.02	0.86±0.05	0.82±0.04	0.87±0.02	0.89±0.02	0.82±0.03
k_1	0.91±0.11	1.23±0.13	1.31±0.09	1.59±0.18	4.04±0.27	2.27±0.09	2.66±0.16
k_2	3.24±0.12	6.12±0.16	5.86±0.17	5.04±0.15	12.31±0.23	12.54±0.21	13.71±0.26
$k_{2,eff}$	1.13±0.04	2.14±0.06	2.05±0.07	1.76±0.05	4.31±0.08	4.39±0.07	4.80±0.09
k_3	0.019±0.0055	0.015±0.005	0.063±0.021	0.016±0.004	0.126±0.02	0.124±0.02	0.069±0.005
q_3	0.0047±0.0006	0.0036±0.0004	0.010±0.002	0.0034±0.0004	0.018±0.003	0.016±0.002	0.015±0.001

Rate constants presented in the table are defined below.

Table 1S shows that the fraction, $f30S_A$, of active $30S_A$ PICs for the best fit of curves in Fig. 5 and Fig. S2 generally exceeds 80%. Comparison with Table 2 in the main text shows that $k_{2,eff}$ corresponds very well with k_{max} values in Table 2 obtained from Linewaver-Burk plots in Fig. 5F. The values of k_{max}/K_M in Table 2 were, however, smaller than the association rate constant k_1 for Met-tRNA$_i$ binding especially in the case of the A1 IF2 mutant. At the same time, in general k_1 and $k_{2,eff} = k_2[50S_0]$ correlated well with the corresponding k_{max}/K_M and k_{max} values for the different IF2 mutants in Table 2.

We have also tried to fit experimental data to a kinetic model in which inactive $30S_I$ PICs do not transform into active 30S PICs but, instead, bind 50S subunits directly with the second order rate constant k_3. The fraction, $f30S_A$, of active 30S PICs in the initial population of 30S PICs was also a fitting parameter of the model along with the three rate constants k_1, k_2 and k_3. Fitting the 70S formation kinetics to this model (Fig. S4C) gave very similar values for k_1 and k_2 rate constants and a similar initial fraction of active 30S

PICs as for the model in Fig. S4B (not shown) indicating that the values of k_1 and k_2 were robust to model choice.

D. Dependence of total protein synthesis in the cell on initiation time and mRNA concentration

The loading rate, V_i, of ribosomes onto mRNA according to the scheme

$$mRNA + 30S \xrightarrow{k_a[30S]} C_1 \xrightarrow{1/\tau_{70S}} C_2 \xrightarrow{1/\tau_{clear}} \quad \text{(D1)}$$

is given by

$$V_i = \left(\tau_{reg} + \tau_{free}\right)^{-1} = \left(\tau_{70S} + \tau_{clear} + \frac{1}{k_a[30S]}\right)^{-1} \quad \text{(D2)}$$

Here, [*30S*] is the concentration of fee 30S subunits and k_a is the rate constant for 30S subunit association to the ribosomal binding site (RBS) on mRNA. We define regeneration time, τ_{reg}, of the RBS of mRNA as:

$$\tau_{reg} = \tau_{70S} + \tau_{clear} \quad \text{(D3)}$$

The term τ_{70S} is the average time for 70S initiation complex formation after binding of a 30S subunit to the mRNA. The term τ_{clear} is the average time for a 70S ribosome to move far enough in the open reading frame (ORF) after 70S initiation complex formation to allow for the binding of a new 30S subunit to the mRNA. The term $1/\left(k_a[30S]\right)$ on the right side of the equation for V_i is the average time during which the regenerated RBS of an mRNA, already competent for 30S subunit binding, remains free. If queuing of ribosomes as they move in the ORF of the mRNA is neglected, the loading rate, V_i, is equal to the rate of initiation of translation per mRNA and has Michaelis-Menten characteristics, as previously shown for initiation of transcription (Bremer et al., 2003; Dennis et al., 2009; Ehrenberg et al., 2009):

$$V_i = \frac{V_i^{\max}[30S]}{K_M + [30S]} \quad \text{(D4)}$$

The maximal initiation rate is given by

$$V_i^{\max} = 1/\tau_{reg} \quad \text{(D5)}$$

The K_M value is given by

$$K_M = 1/(k_a \tau_{reg}) \quad \text{(D6)}$$

The total rate, V_p, of protein synthesis per cell volume, proportional to the growth rate of the cell population (Ehrenberg and Kurland, 1984), is given by:

$$V_p = [mRNA_0] \cdot N_r v_e \quad \text{(D7)}$$

Here, $[mRNA_0]$ is the total mRNA concentration in the cell, N_r is the average number of translating ribosomes per mRNA and v_e is the average rate of protein elongation per ribosome. N_r can be obtained from the average time, τ_{transl}, to translate all codons in the ORF and the initiation time τ_{init} (or the initiation rate V_i) through (Dennis et al., 2009):

$$N_r = \frac{\tau_{transl}}{\tau_{initi}} = V_i \tau_{transl} \quad \text{(D8)}$$

The total average number of ribosome, N_{tot}, per mRNA (the translating ones plus those in the initiating state C1 in the scheme D1) is given by

$$N_{tot} = \frac{\tau_{70S} + \tau_{transl}}{\tau_{initi}} = V_i(\tau_{70S} + \tau_{transl}) \quad \text{(D9)}$$

The maximal total average number, $N_{tot}^{\max}$ of ribosome per mRNA is obtained when the initiation rate V_i is maximal. Using relation (D5) one obtains:

$$N_{tot}^{\max} = V_i^{\max}(\tau_{70S} + \tau_{transl}) = \frac{\tau_{70S} + \tau_{transl}}{\tau_{reg}} \quad \text{(D10)}$$

The average protein translation time is given by

$$\tau_{transl} = n_c / v_e \quad \text{(D11)}$$

The total rate of protein synthesis per cell volume can now be explicitly written as

$$V_p = [mRNA_0] \cdot N_r v_e = \frac{n_c}{\tau_{reg}}[mRNA_0]\frac{\tau_{reg}k_a[30S]}{1+\tau_{reg}k_a[30S]} \quad \text{(D12)}$$

The concentration of translating ribosomes, $[R_{transl}]$, is given by:

$$[R_{transl}] = [mRNA_0] \cdot N_r = \frac{n_c}{v_e}[mRNA_0]\frac{k_a[30S]}{1+\tau_{reg}k_a[30S]} \quad \text{(D13)}$$

To determine the concentration of the free 30S subunit, we recall that the free 30S concentration plus the concentration of 30S subunit bound to mRNA either as subunit or in 70S ribosomes equals the total concentration of the 30S subunits, $[30S_0]$ in the cell:

$$[30S_0] = [30S] + [mRNA_0] \cdot N_{tot}$$

Taking into account relations (D9) for N_{tot} and (D2) for V_i one gets:

$$[30S_0] = [30S] + [mRNA_0]\frac{(n_c/v_e + \tau_{70S})k_a[30S]}{1+\tau_{reg}k_a[30S]} \quad \text{(D14)}$$

This quadratic equation shows that the concentration $[30S]$ of the free 30S subunit is determined by the total concentration of 30S subunit $[30S_0]$ and mRNA concentration $[mRNA_0]$ in the cell. Together, relations (D12) and (D14) determine how V_p depends on $[mRNA_0]$ and $[30S_0]$ for any choice of the parameters τ_{70S}, τ_{clear}, k_a, n_c, v_e, as exemplified in Fig. S5 for realistic values for these parameters as discussed in the main text (see the legend of Figure S5 for more details).

mRNA-limited protein synthesis. In the limiting case, where $\tau_{reg}k_a[30S] >> 1$, relation (D12) shows that the total rate of protein synthesis per cell volume is given by

$$V_p = [mRNA_0] \cdot n_c / \tau_{reg} \tag{D15}$$

This defines the condition of mRNA limitation, where V_p is proportional $[mRNA_0]$ and inversely proportional to τ_{reg}. In addition, in this limit relation (D14) is approximated by:

$$[30S] = [30S_0] - [mRNA_0]\frac{(n_c / v_e + \tau_{70S})}{\tau_{reg}}$$

(D16)

The ratio $\frac{(n_c / v_e + \tau_{70S})}{\tau_{reg}}$ in relation (D16) defines, according to (D10), the maximal number of ribosomes on an mRNA with n_c codons. Taking into account that $\tau_{reg} = \tau_{70S} + \tau_{clear}$ it follows that under the condition of mRNA limitation and if $\tau_{70S} >> \tau_{clear}$ the average number of ribosomes on mRNA is inversely proportional and the ribosome density in polysomes is proportional to the time, τ_{70S}, of 70S initiation complex formation. From relation (D16) it follows that the condition $\tau_{reg}k_a[30S] >> 1$ for mRNA-limited protein synthesis requires that

$$[mRNA_0] < \frac{\tau_{reg}}{(n_c / v_e + \tau_{70S})}[30S_0] \tag{D17}$$

Our model calculations (Figure S5) show that expression (D17) approximates the region for mRNA-limited protein synthesis even for k_a=1 $\mu M^{-1}s^{-1}$, a value considerably below experimental estimates of the rate constant for association of 30S subunits to the RBS of mRNAs (Studer and Joseph, 2006).

Ribosome-limited protein synthesis. When the mRNA concentration increases above:

$$[mRNA_0] > \frac{\tau_{reg}}{(n_c / v_e + \tau_{70S})}[30S_0], \tag{D18}$$

the concentration of free 30S subunits decreases dramatically, so that the condition $\tau_{reg}k_a[30S] >> 1$ for mRNA limitation breaks down. This follows from (D13), showing that otherwise the number of mRNA bound ribosomes will exceed the total number of ribosomes in the cell. According to (D6), the physiologically relevant condition $\tau_{reg}k_a[30S_0] >> 1$ implies that the total concentration of 30S subunits is much larger than the K_M-value for initiation of protein synthesis, *i.e.*:

$$[30S_0] >> \frac{1}{k_a \tau_{reg}} = K_M \tag{D19}$$

This condition is satisfied even for unrealistically small k_a-values ~1 μM^{-1}s^{-1} (see above), since the inequalities τ_{reg} > 1 s (see the main text) and [30S_0]>>1 μM are valid. In the mRNA region defined by (D18) with $k_a[30S]\tau_{reg} = w \approx 1$, it follows from (D6) that:

$$[30S] = \frac{[30S_0]}{1 + \frac{\tau_{reg} k_a}{1+w}[mRNA_0]\frac{(n_c/v_e + \tau_{70S})}{\tau_{reg}}} < \frac{[30S_0]}{1 + \frac{1}{1+w}(\tau_{reg} k_a)[30S_0]} << [30S_0]$$

The first inequality in this relation follows from (D18) and the second from (D19). Given that [30S]<<[30S_0], (D14) simplifies to:

$$[30S_0] \approx [mRNA]\frac{(n_c/v_e + \tau_{70S})k_a[30S]}{1 + \tau_{reg} k_a[30S]},$$

which can be rewritten as

$$\frac{[30S_0]}{(n_c/v_e + \tau_{70S})} \approx [mRNA]\frac{k_a[30S]}{1 + \tau_{reg} k_a[30S]} \qquad \text{(D20)}$$

Substituting (D20) into (D12) gives the total rate of protein of protein synthesis as:

$$V_p = v_e[30S_0]\frac{1}{(1 + \tau_{70S} v_e / n_c)} \qquad \text{(D21)}$$

This is the condition of ribosome limitation, where protein synthesis does not depend on the mRNA concentration, but only on the total ribosome concentration. This approximation holds well for realistic parameter values already when the total mRNA concentration is just above the boundary set by inequality (D17) for mRNA-limited protein synthesis (Fig. S5). Furthermore, the transition between mRNA-limited and ribosome-limited protein synthesis becomes sharper with increasing k_a values, also illustrated in Fig. S5.

Figure S5 also shows that the maximal difference in generation times (maximal ratio of generation times) between the wild type and *fmt-* strains is observed for mRNA concentrations where protein synthesis is mRNA-limited in both wild type and mutant strain. From (D15) follows that in this case the ratio between generation times is given by:

$$\frac{\tau_{clear} + \tau_{70S}(fmt - mutamt)}{\tau_{clear} + \tau_{70S}(wild - type)} = \frac{1.2s + 2.8s}{1.2s + 0.25s} \approx 2.76 \qquad \text{(D22)}$$

The ratio between generation times decreases gradually as the total mRNA concentration increases above the boundary for the mRNA-limited protein synthesis in the wild type strain and is minimized when protein synthesis is ribosome-limited for both wild type and *fmt-* strain. From (D21) follows that the ratio between generation times under ribosome limitation is given by:

$$\frac{n_c/v_e + \tau_{70S}(fmt - mutant)}{n_c/v_e + \tau_{70S}(wild - type)} = \frac{20s + 2.8s}{20s + 0.25s} \approx 1.13 \qquad \text{(D23)}$$

References

Altman, E., Roth, J.R., Hessel, A. and Sanderson, K.E. (1996) Transposons Currently in Use in Genetic Analysis of Salmonella Species. In Neidhardt, F.C. and Curtiss, R. (eds.), *Escherichia coli and Salmonella Typhimurium: Cellular and Molecular Biology*. American Society for Microbiology, Washington, D.C., Vol. 2, pp. 2613–2626.

Antoun, A., Pavlov, M.Y., Lovmar, M. and Ehrenberg, M. (2006) How initiation factors tune the rate of initiation of protein synthesis in bacteria. *Embo J*, 25, 2539-2550.

Antoun, A., Pavlov, M.Y., Tenson, T. and Ehrenberg, M.M. (2004) Ribosome formation from subunits studied by stopped-flow and Rayleigh light scattering. *Biol Proced Online*, 6, 35-54.

Bremer, H., Dennis, P. and Ehrenberg, M. (2003) Free RNA polymerase and modeling global transcription in Escherichia coli. *Biochimie*, 85, 597-609.

Dennis, P.P., Ehrenberg, M., Fange, D. and Bremer, H. (2009) Varying rate of RNA chain elongation during rrn transcription in Escherichia coli. *J Bacteriol*, 191, 3740-3746.

Ehrenberg, M., Dennis, P.P. and Bremer, H. (2009) Maximum rrn promoter activity in Escherichia coli at saturating concentrations of free RNA polymerase. *Biochimie*.

Ehrenberg, M. and Kurland, C.G. (1984) Costs of accuracy determined by a maximal growth rate constraint. *Q Rev Biophys*, 17, 45-82.

Milon, P., Konevega, A.L., Gualerzi, C.O. and Rodnina, M.V. (2008) Kinetic checkpoint at a late step in translation initiation. *Mol Cell*, 30, 712-720.

Studer, S.M. and Joseph, S. (2006) Unfolding of mRNA secondary structure by the bacterial translation initiation complex. *Mol Cell*, 22, 105-115.
